# The semiconstrained DRUJ prosthesis: blessing or curse?

**DOI:** 10.1007/s00068-023-02304-x

**Published:** 2023-07-26

**Authors:** Katharina Sommer, Ramona Sturm, Jasmina Sterz, Ingo Marzi, Johannes Frank

**Affiliations:** Department of Trauma, Hand and Reconstructive Surgery, Goethe University Frankfurt, Theodor Stern Kai 7, 60590 Frankfurt Am Main, Germany

**Keywords:** DRUJ, Aptis semi constrained prosthesis, DRUJ instability, DRUJ arthrosis

## Abstract

**Purpose:**

There are numerous operative procedures to treat osteoarthritic changes or a significant instability of the distal radioulnar joint (DRUJ). The key problem of most methods is the destabilization of the forearm leading to secondary painful impingement between the radius and ulna, as well as a significant limitation of forearm rotation. The Aptis-Prosthesis designed by Scheker represents a complete substitute for the DRUJ. It is mostly used after the failure of various treatment options to solve the primary problems (arthritis, instability). We have used this type of prosthesis mostly after multiple operative treatments for more than 25 years.

**Methods:**

In the following retrospective study, we analyzed the data of patients that received an Aptis-prosthesis between 2016 and 2021. We have implanted this prosthesis in 13 cases (11 female, 2 male). Routinely, we document the clinical outcome concerning range of motion (ROM), grip strength, and pain according to numeric rate scaling (NRS) after more than 12 months (month 12–24). In addition, complications, osseous changes, and the rate of loosening of the prosthesis were registered. Furthermore, DASH-Score and patients ‘ satisfaction were evaluated. Also—as with other implants—follow-up x-rays were performed.

**Results:**

Removal or significant revision of any of the prostheses was not needed. The ROM was 68.1° ± 19.7° for pronation and 72.3° ± 20.9° for supination, grip strength amounted to 27.7 kg ± 11.0 kg equaling 83% of the contralateral side. NRS was 0 at rest and 1.2 (0–2) under weight-bearing. A lysis margin of the radial tap was noted in the radiological examination in 2 patients but without any signs of loosening. The DASH-Score added up to 31.8 ± 13.8 (13–55). All patients were satisfied or very satisfied having this implant.

**Conclusion:**

The semiconstrained Aptis-prosthesis is a safe and efficient treatment option after failed DRUJ surgeries. It is striking that of the 20 implanted prostheses no significant revision or explantations were necessary over a period of 25 years.

## Introduction

Instability and arthrosis of the distal radioulnar joint (DRUJ) is a serious problem in patients causing loss of function of the hand by limiting load bearing as well as loss of motion at the wrist, as a result of either posttraumatic or congenital changes, rheumatoid or inflammatory arthritis, tumors or degenerative instabilities [1, 2].

The treatment of this condition is difficult due to the complex anatomy of the DRUJ [2]. The ulna is the firm column of the forearm with the radius rotating around it [3]. Furthermore, for lifting, the ulna head has to support this function to stabilize the radius in the sigmoid notch together with the structures of the triangular fibrocartilage complex (TFCC) [3].

In cases of persisting instability, salvage procedures as Sauvé-Kapandji, Darrach, or Bowers are often chosen to ameliorate this situation. But in the long run, they are likely to result again in instability and a painful impingement between the radius and ulna, leading to a loss in the range of motion as well as limited load bearing of the arm [4–7].

**Fig. 1:**
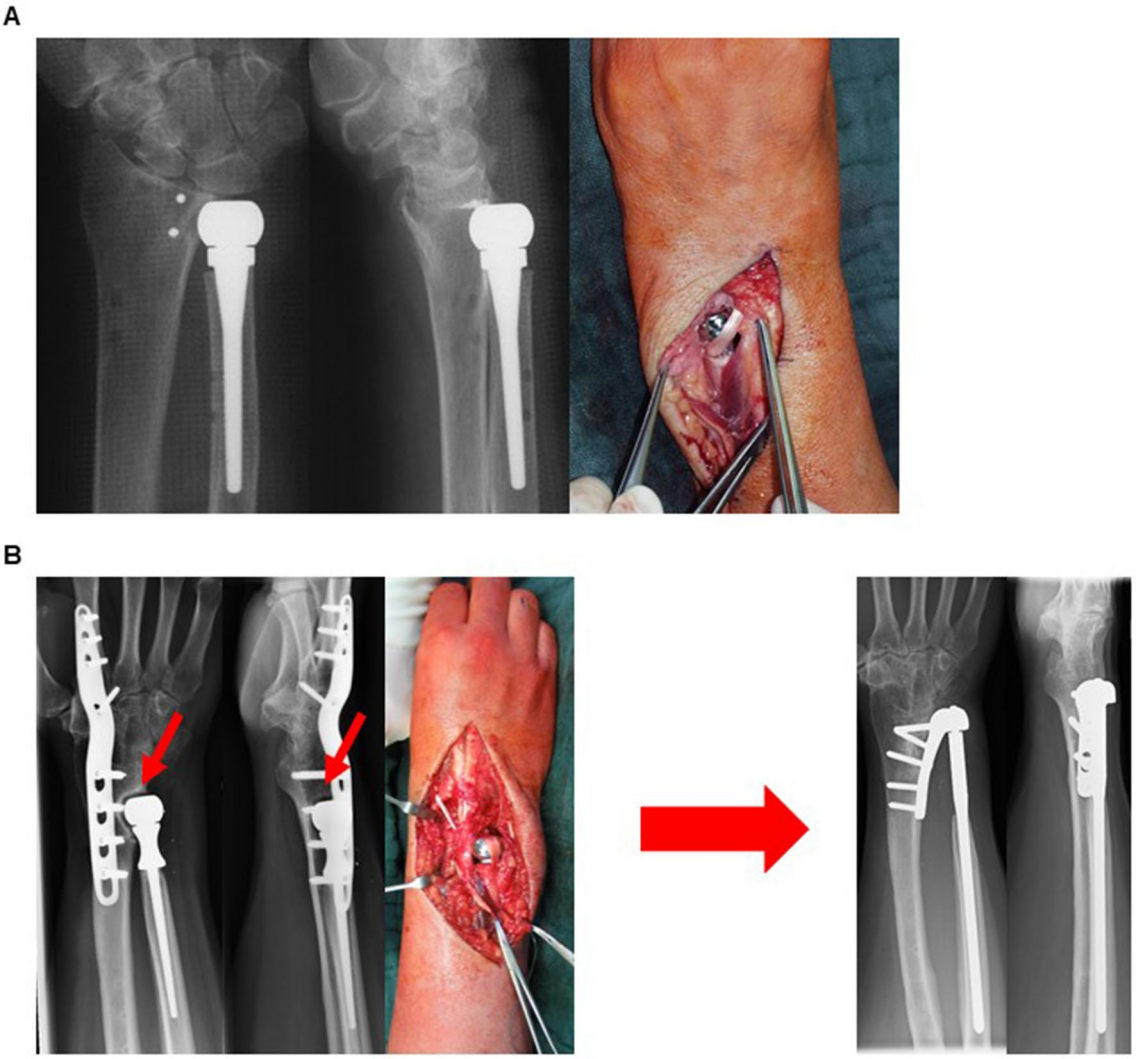
Radiograph of an ulna head prosthesis with intraoperative pictures of the ligament reconstruction using a tendon graft to replace the lost stabilizers. Radiograph of an ulna head prosthesis 3 years after implantation significantly gouging into the distal radius causing pain and instability, on the right side condition after implantation of an Aptis DRUJ prosthesis

There are still several different models to replace the ulna head on the market. Up to now, the most common option is a simple replacement of the ulna head alone mostly combined with a reconstruction of the ligaments (e.g. UHP, Martin GMBH, Germany, U-Head, Stryker Corporation (NYSE:SYK), USA” prostheses, former U-Head, Small Bone Innovation, Fig. 1A). The second option is a semi constrained prosthesis that additionally uses a radial implant to stabilize the DRUJ to avoid ulna instability (Schuurman AH DRUJ prosthesis).

A more recently invented procedure is a prosthesis that is implanted in the ulna osteotomy as performed for a Sauvé-Kapandji procedure [8]. This stabilizes the ulna stump allowing a full range of motion by rotation due to the prosthesis in the area of the osteotomy (Table 1).

**Table 1:** Various DRUJ-Implants

Constrained implants	Non-constrained implants
Moradi intraosseous prosthesis	First choice DRUJ system, integra partial/total head replacement
Schuurman AH DRUG prosthesis	U-Head, Stryker (SBI)
Aptis DRUJ prosthesis	UHP, Martin GmbH

Most studies only evaluate the range of motion after surgery, neglecting the pivotal role of the ulna in lifting weights. Implanting hemiprosthesis or a simple replacement of the ulna will lead to an impairment in lifting or cause grouting into the radius and DRUJ instability [9].

Since we have many years of experience in implanting the Aptis prosthesis as a rescue option for failed salvage procedures, as well as failed ulna head replacements, we present our new mid-term outcome regarding the postoperative range in motion, grip strength, patient satisfaction, DASH, numeric rating for pain (NRS), osseous alterations in the radiograph, and complications.

## Patients and methods

In the period between 2016 and 2021 we implanted 13 Aptis DRUJ-Prostheses in 13 patients. After permission from the institutional review board (GN2022-939) we retrospectively analyzed the clinical data of these patients. Inclusion criteria were the implantation of an Aptis DRUJ-Prosthesis during this time. Exclusion criteria were continued treatment in another clinic. There was no patient excluded.

11 of the patients were female (84.6%) and subsequently 2 male (Table 2). 9 prostheses were implanted on the right side, thus 4 on the left side (Table 2). The average age of the patients on the day of implantation was 43.9 ± 13.7 standard deviation (SD) years ranging from 22 to 59 years (Table 2).

**Table 2:** Patients

Age (years)	43.9 ± 13.7
*Gender*	
Female	11
Male	2
*Operated side*	
Right	9
Left	4
*Indication for Aptis prosthesis*	
Chronic instability/arthrosis	5
Posttraumatic	4
Congenital deformity	2
After common ulna head replacement	2

The clinical and radiological follow-up took place during a routine checkup at least after 12 months and the longest after 24 months after implantation of the prosthesis.

For evaluation, we registered indications, complications, range of motion, strength and pain according to the numeric rate scaling (NRS). The radiographs were evaluated for signs of osseous alterations or loosening of the prosthesis. Furthermore, we evaluated patients’ satisfaction with the result of the surgery as well as the DASH-Score.

Statistical analysis was performed with a non-parametric Wilcoxon-Mann–Whitney-*U*-Test using BiAS 10.06.

### Surgical technique

**Fig. 2:**
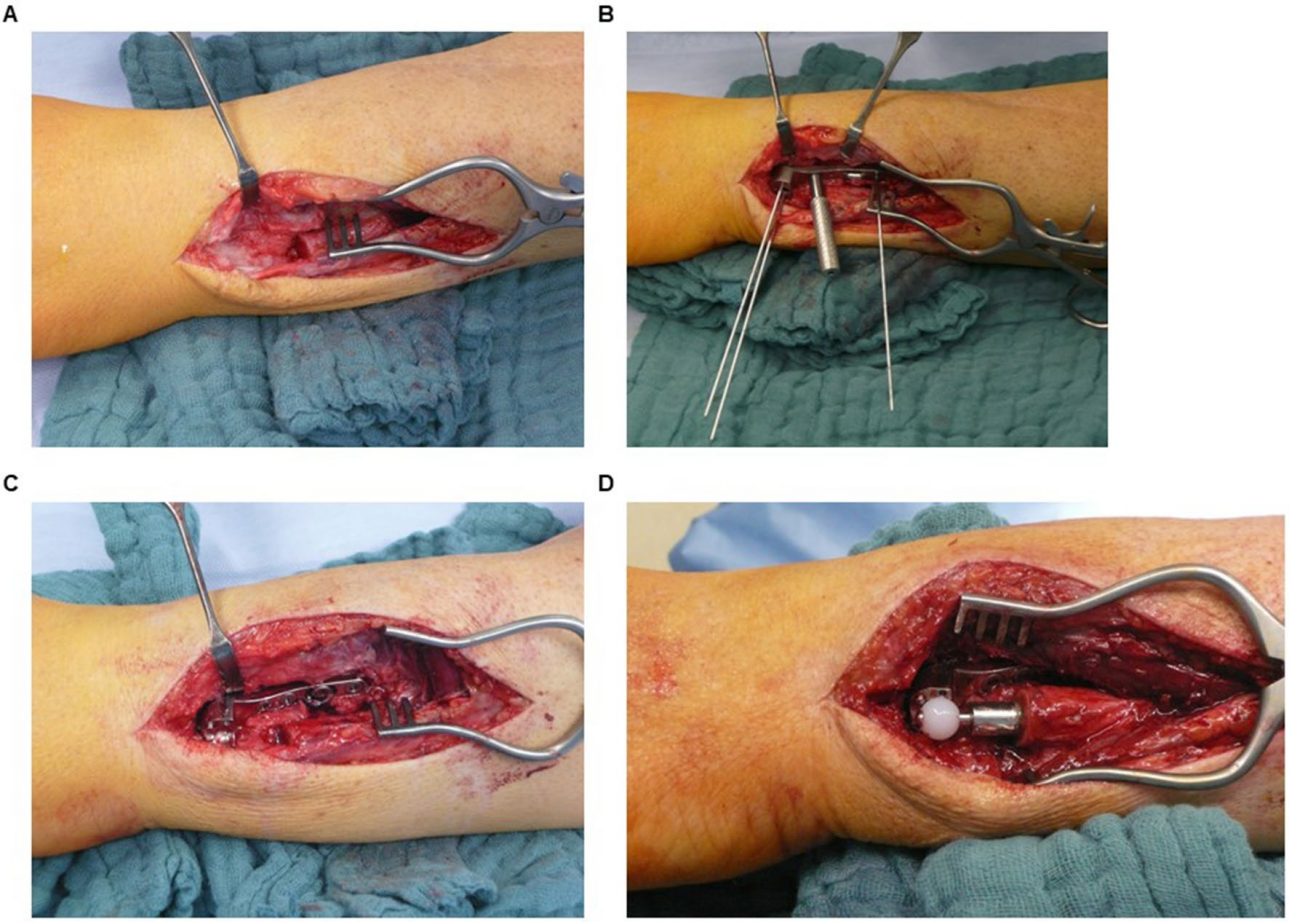
Surgical procedure. Picture of the prepared ulna after resection of the head distally. Fitting of the template to the radius with wires for allocating the position of the PEG hole. Fixation of the radial implant with 3.5 mm screws. Implantation of the ulna stem and head after reaming the shaft

**Fig. 3:**
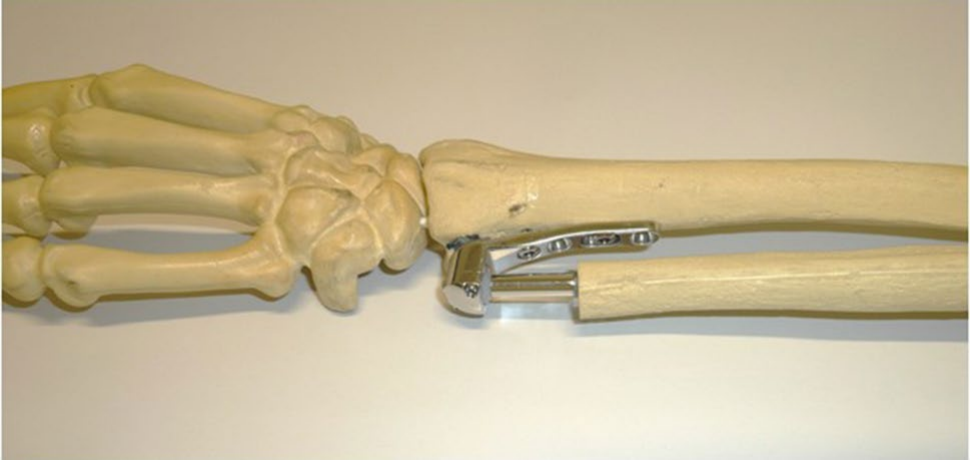
Picture of an artificial bone with the implanted APTIS semiconstrained DRUJ prosthesis

The detailed technique has already been published elsewhere [9]. After skin incision and mobilization of the extensor tendons the ulna head is resected distally (Fig. 2A). Then a template is fitted to the radius and temporarily fixed with k-wires (Fig. 2B). After image verification of the correct positioning, the PEG guiding is drilled, and the size-adapted radial implant is fixed with 3.5 mm screws to the bone (Fig. 2C). After reaming of the ulna, the length-adapted ulna stem is implanted (Fig. 2D). The ulna head is adjusted and secured after repositioning by fastening the metallic lid to the radial implant with a screw. After x-ray control and rinsing, the wound is closed (Fig. 3).

The arm is then immobilized with an upper arm splint depending on the patient’s pain level. Supervised mobilization is started about 1 week after surgery.

## Results

**Fig. 4:**
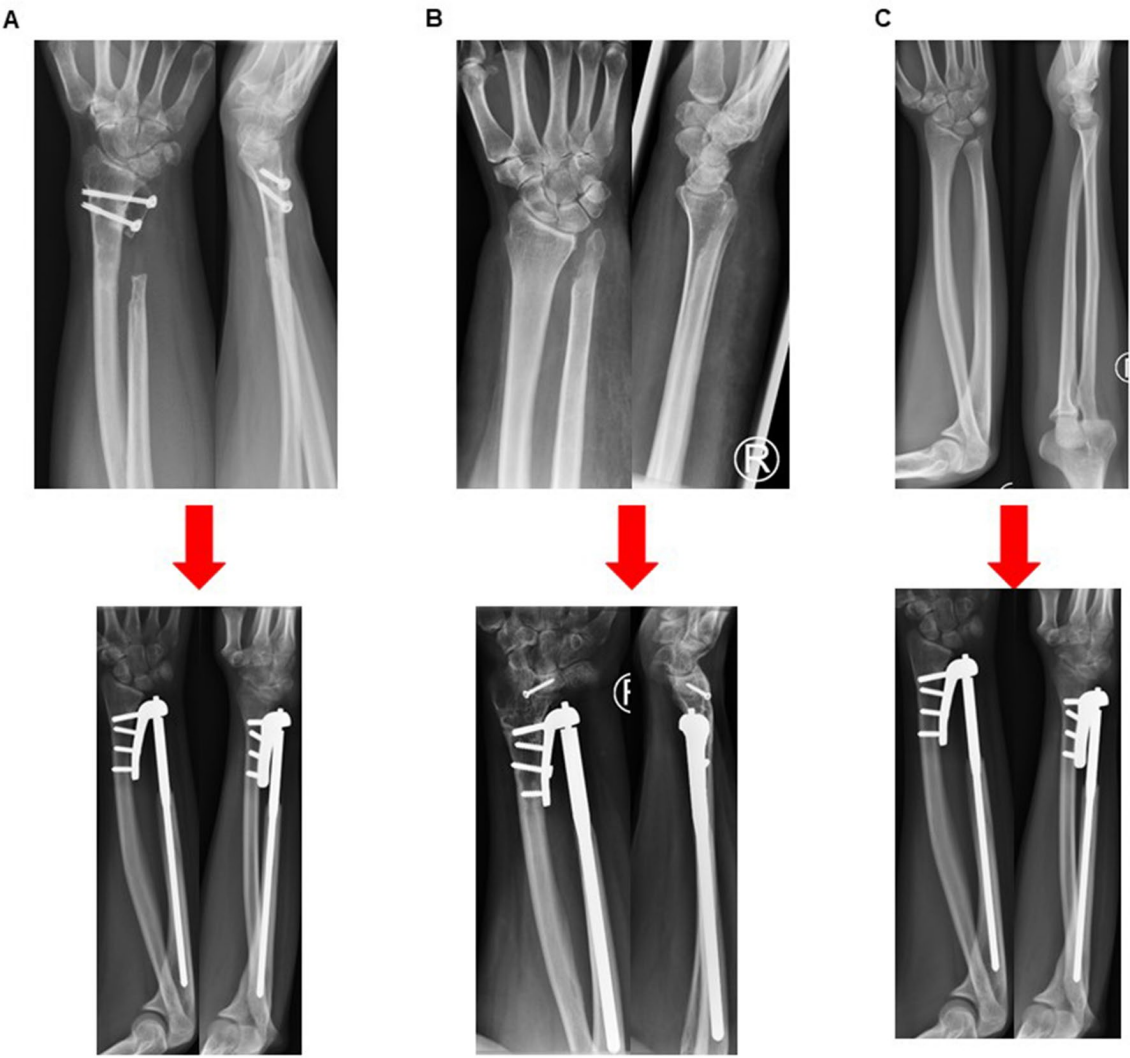
Radiograph of a wrist after Sauvé-Kapandji procedure fusing the ulna head to the distal radius and resecting a part of the ulna shaft to still enable rotation in the forearm, on the bottom pictures after implantation of an Aptis DRUJ prosthesis. Radiograph of a wrist after Bowers procedure with hemiresection of the ulna head and interposition arthroplasty on the bottom pictures after implantation of an Aptis DRUJ prosthesis. Radiograph of an acquired or congenital deformity of the proximal radioulna joint causing DRUJ pain on the bottom pictures after implantation of an Aptis DRUJ prosthesis

Most common indication for implantation was chronic instability and arthrosis of the DRUG in 5 patients followed by posttraumatic deformities in 4 patients (Table 2). In 2 patients, implantation was due to complications after implantation of an ulna head prosthesis and 2 patients had a congenital deformity. All but 1 patient underwent previous surgeries on the DRUJ before the implantation of the Aptis prosthesis (Table 3; Fig. 4A–C).

**Table 3:** Results

Number of previous surgeries	2.7 ± 2.0SD (0–7)
Revisions	0
Complications	1
numbness	1
*Range of motion*	
Pronation	68.1° ± 19.7°SD
Supination	72.3° ± 23.9°SD
Force	27.7 kg ± 11.0 kg SD
*NRS*	
At rest	0
Under weight bearing	1.2 (0–2)
*Radiologic outcome*	
Lysis around the radial tap	2
DASH	31.8 ± 13.8
*Patients satisfaction*	
Very satisfied	8
Satisfied	5

Average number of previous surgeries on the same wrist was 2.7 ± 2.0 SD ranging from 0 to 7 (Table 3). There was no complication that had to be revised by surgery after implantation of the DRUJ-Prosthesis. No intervention was needed due to infection or loosening of the prosthesis. No prosthesis had to be removed. A primary implantation was chosen due to congenital deformity and instability of the DRUJ.

The average range of motion was 68° ± 20° SD (20° minimum (min), 80° maximum (max)) for pronation and 72° ± 24° SD (20°min, 90°max) for supination (Table 3). Grip strength averaged 28 kg ± 8 SD (14 kg min 40 kg max). This means 83% strength (76–100%) compared to the uninjured opposite side (Table 3). This difference was not significant with a p value of 0.10.

The pain level measured by NRS was 0 points at rest and in average 1.2 points (0 min, 2 max) under weight-bearing (Table 3).

On the radiographs, a lysis margin of the radial tap was noted in 2 patients (Table 3). Nevertheless, there were no signs of loosening of the prosthesis and no other osseous changes were noted. There was only one minor complication, as numbness of the dorsal side of the hand was reported by one patient. Especially, no surgical revision had to be performed even in the patients with the lysis margin of the radial tap.

DASH-Score measurements revealed 32 ± 14 SD points (13 min to 55 max). 5 patients were satisfied and 8 were very satisfied with the result of the surgery (Table 3).

## Discussion

In our present study, we evaluated the mid-term and long-term results after the implantation of a Total- DRUJ prosthesis to salvage significant congenital deformities or failed DRUJ surgery.

The distribution between the gender varies from study to study. We found that most of the 13 treated patients were female with 84.6% (11) and just 15.4% male (2). In Schuuhmann’s study in the Netherlands, the distribution was 2 male and 17 female patients, equally to ours [10]. However, in the two studies by Lans and Savvidou, both conducted in the USA, the gender distribution was much more balanced: 21 females and 14 males in the first study and 7 males and 7 females in the second [2, 10]. This high variance in gender distribution might be caused by the low number of patients treated in all studies.

The average age of the patients in our group amounted to 43.7 years which is about the same range as some younger patients as in most of the other studies [2, 10, 11]. Interestingly, for the salvage procedures the average age is mostly performed in older patients compared to the average age of our study [12, 13].

Regarding the grip strength, Kakar et al. achieved a worse result after implantation of the DRUJ prosthesis with 52% of the strength of the opposite healthy side compared to our results pf 83%. The results of Savvidou et al. are more similar to ours, as patients reached a grip strength of 90% of the contralateral side [2, 14].

Schuurman reached with the prosthesis designed by himself a grip strength of 16 kg, which is less than usually achieved with the Aptis prosthesis e.g. 26 kg in the study of Lans et al. [10, 15]. Unfortunately, for the intra-osseous Kapandji extension prosthesis no long-term results for grip strength are available right now so an advantage or disadvantage compared to the Aptis prosthesis still has to be investigated [3].

Concerning the DASH score, patients displayed a median score of 32 ± 14 SD points. Brannan et al. reached slightly better results with a score of 26.7 after the same procedure [16]. Mehling et al. accomplished worse results with an ulna head prosthesis by Herbert with patients displaying a median score of 43 [17].

Lately, Pääkköken and Bellevue et al. described a high rate of revisions and complications with the Aptis prosthesis (60%) [11, 18]. This is very surprising, as in our own hands, we had just one revision in all of the 20 prostheses implanted and none in the present study examining the latest 13 patients. In their series, Warlop et al. described a reoperation rate of 24% with an overall survival rate of 92% of the prostheses [19]. This result is shared with Brannan et al., who found the same rate of reoperations [16]. The success of the prosthesis thus seems to depend significantly on the correct implantation and experience of the executing surgeon [19]. Savvidou discusses in detail the essential steps to avoid complications that are achieved by careful tissue handling and correct implantation [2].

Especially, the horizontal drilling in the direction of the 1st Extensor compartment to implant the radial component must be done carefully, because the superficial radial nerve is at risk as well. Therefore, the ankle-stable new screws have to be chosen short enough to easily avoid this problem. With respect to the soft tissue complications, it should be noted, that in reoperations the ligamentous structures are diminished and therefore especially the tendinosis of the ECU might persist. Due to the significant complaints and pain before implantation, we can conclude from our experience that this might persist to some degree, however, in our patients it resolves, especially with the new smaller design of the Aptis Prosthesis.

Of course, this study is limited by the low number of patients included, as there is only a small number to report on and on the retrospective design.

Nevertheless, in our opinion the semi constrained Aptis DRUJ prosthesis is a safe and efficient treatment option after failed DRUJ surgery and might even be considered as the first option in severe deformities. Striking is the fact, that of the 20 implanted prostheses up to now no exchange of parts or explantations of the prostheses was needed.

## Data Availability

The data sets used and analyzed in the current study are available from the corresponding author upon reasonable request.
